# Supplementary material for: Content-rich biological network constructed by mining PubMed abstracts
Source: BMC Bioinformatics. 2004 Oct 8;5:147. doi: 10.1186/1471-2105-5-147 (PMC528731; doi:10.1186/1471-2105-5-147)
Supplement: Additional File 2 — The original results of the above study (non-essential files are deleted to keep the file size under the limit set by BMC bioinformatics). [file 1471-2105-5-147-S2.bz2 › chilibotAdditionalFile2/dip50/79ID9033409E291/html/CDC42_PAK3.html]

 


 **CDC42** and **PAK3** 
  
Found 226 abstracts in PubMed, retrieved 50.  
 

 What does Google say? 
 PDF only 
| .edu only 

---

**Interactive relationship** (e.g. stimulation, inhibition, etc)

**Neutral relationship**- Furthermore, overproduction or loss of Rga1 and Rga2, but not Bem3, affected the two hybrid interaction of  **Cdc42**  with Ste20, a p21  [ **PAK3** ]  activated kinase kinase required for haploid invasive growth.  Ref: 12455995 Eukaryot Cell, 2002
- p21  [ **PAK3** ]  Activated kinases are regulated by the GTPases Rac and  **Cdc42**  and control actin dynamics and phosphorylation of the oxidase component p47.  Ref: 12189148 J Biol Chem, 2002
- The immunoprecipitants from bovine brain homogenate with anti PAK1 and  **PAK3**  antibodies incorporated P into synapsin I in a  **Cdc42**  GTPgammaS dependent manner, and its phosphorylation site was confirmed as Ser using pS603 Syn I Ab.  Ref: 12237306 J Biol Chem, 2002
- The p21  [ **PAK3** ]  activated protein kinases are regulated by the Rho family GTPases Rac and  **Cdc42** .  Ref: 12529371 J Biol Chem< MedlineTA>J Biol Chem, 2002
- Mammalian p21  [ **PAK3** ]  activated kinase 1 Pak1 is a highly conserved effector for the small GTPases  **Cdc42**  and Rac1.  Ref: 12176333 Curr Biol, 2002
- p21  [ **PAK3** ]  activated kinase 1 PAK1 is an effector of Rac  **Cdc42**  GTPases that has been implicated in the regulation of cytoskeletal dynamics, proliferation, and cell survival signaling.  Ref: 12522133 J Biol Chem, 2003
- Concomitant with our finding that the small GTPases  **Cdc42**  and Rac1 were activated by attachment to FN, constitutively active ca  **Cdc42**  and ca Rac1 promoted down regulation of p21  [ **PAK3** ]  CIP1.  Ref: 12052868 Mol Cell Biol, 2002
- Two closely related p21  [ **PAK3** ]  activated kinases from Saccharomyces cerevisiae, Ste20 and Cla4, interact with and are regulated by  **Cdc42** , a small Rho like GTPase.  Ref: 12588977 Mol Cell Biol, 2003
- The family of p21  [ **PAK3** ]  activated kinases PAKs have been implicated in the rearrangement of actin cytoskeleton by acting downstream of the small GTPases Rac and  **Cdc42** .  Ref: 12181358 Mol Biol Cell, 2002
- p21  [ **PAK3** ]  activated protein kinases PAK are a family of serine threonine protein kinases which are activated by binding of the p21  [ **PAK3** ]  G proteins  **Cdc42**  or Rac.  Ref: 12853446 J Biol Chem< MedlineTA>J Biol Chem, 2002
- The p21  [ **PAK3** ]  activated protein kinases Paks are serine threonine protein kinases activated by binding to Rho family small GTPases, Rac and  **Cdc42** .  Ref: 12860998 J Biol ChemJ Biol Chem, 2002
- However, dominant negative dn  **Cdc42**  and dn Rac1 mutants blocked the anchorage induced degradation of p21  [ **PAK3** ]  CIP1, suggesting that an integrin induced  **Cdc42**  Rac1 signaling pathway activates proteasomal degradation of p21  [ **PAK3** ]  CIP1.  Ref: 12052868 Mol Cell Biol, 2002
- The p21  [ **PAK3** ]  activated kinases are serine threonine protein kinases that bind to and, in some cases, are stimulated by activated forms of the small GTPases,  **Cdc42**  and Rac.  Ref: 11950587 Int J Biochem Cell Biol, 2002

**Non-interactive relationship** (e.g. studied together, co-existance, homology, etc.)

- **Cdc42**  regulation of kinase activity and signaling by the yeast p21  [ **PAK3** ]  activated kinase Ste20.  Ref: 11940652 Mol Cell Biol, 2002
- Cell attachment to the extracellular matrix induces proteasomal degradation of p21  [ **PAK3** ]  CIP1 via  **Cdc42**  Rac1 signaling.  Ref: 12052868 Mol Cell Biol, 2002
- RESULTS We determined if the vimentin filaments can be regulated by p21  [ **PAK3** ]  activated kinase, one of targets downstream of  **Cdc42**  or Rac.  Ref: 11895474 Genes Cells, 2002
- The small GTPase  **Cdc42** , its inhibitory subunit Rho GDI, the adapter protein Nck, gamma p21  [ **PAK3** ]  activated protein kinase, and cofilin were found to undergo rapid association with the developing phagosomes in these cells.  Ref: 12189515 Histochem Cell Biol, 2002
- The Rho like GTPase  **Cdc42** , its GEF Cdc24 and its effector Cla4 a member of the p21  [ **PAK3** ]  activated kinases control the initial binding and activation of Lte1 to the bud cortex.  Ref: 12234925 EMBO J, 2002
- The pathway initiated by m2 receptors involved sequential activation of Gbetagamma i3, phosphatidylinositol PI 3 kinase,  **Cdc42** , and Rac1, p21  [ **PAK3** ]  activated kinase PAK1, p38 mitogen activated protein MAP kinase, and cPLA 2, and phosphorylation of cPLA 2 at Ser 505.  Ref: 12576304 Am J Physiol Gastrointest Liver Physiol, 2003
- The small GTP binding protein  **Cdc42** , the guanine nucleotide exchange factor Scd1, the p21  [ **PAK3** ]  activated kinase Shk1, and the adaptor protein Scd2 are involved in the  **Cdc42**  dependent signaling cascade in fission yeast.  Ref: 12409291 J Biol Chem, 2003
- Mutation of histidine residues 19 and 22 to leucine on the p21  [ **PAK3** ]  binding domain of Pak5 completely abolished the binding of  **Cdc42**  and the  **Cdc42**  mediated autophosphorylation.  Ref: 12860998 J Biol ChemJ Biol Chem, 2003
- The p21  [ **PAK3** ]  activated kinases PAKs 1 3 are serine threonine protein kinases whose activity is stimulated by the binding of active Rac and  **Cdc42**  GTPases.  Ref: 12676796 Annu Rev Biochem< MedlineTA>Annu Rev Biochem, 2003
- In brain, Shank forms a complex with beta PIX and beta PIX associated signaling molecules including p21  [ **PAK3** ]  associated kinase PAK, an effector kinase of Rac1  **Cdc42** .  Ref: 12626503 J Biol Chem, 2003
- Expression of the  **Cdc42**  Rac interacting domain of p21  [ **PAK3** ]  activated kinase in macrophages abolished the p59Hck induced morphological changes.  Ref: 11904303 J Biol Chem, 2002
- Evidence has been provided that direct binding of  **Cdc42**  guanosine triphosphate and Rac1 GTP to the N terminal regulatory domain of the p21  [ **PAK3** ]  activated kinase stimulates PAK activation and actin reorganization.  Ref: 12453877 Blood, 2002
- One of the effectors of Rac1 and  **Cdc42** , p21  [ **PAK3** ]  activated kinase 1, is abundant in the brain.  Ref: 12213441 Mol Cell Neurosci, 2002
- The present study aimed to elucidate the subcellular distribution of the  **Cdc42**  and Rac activated alpha and beta isoforms of p21  [ **PAK3** ]  activated kinase in the monkey cerebral neocortex and hippocampus.  Ref: 12012157 Exp Brain Res, 2002
- The p21  [ **PAK3** ]   **Cdc42**  Rac activated kinase PAK mediates a number of biological effects downstream of these Rho GTPases reviewed by 1.  Ref: 11864573 Curr Biol, 2002
- We describe a signaling mechanism involving Gbetagamma, PAK associated guanine nucleotide exchange factor PIXalpha,  **Cdc42** , and p21  [ **PAK3** ]  activated kinase PAK 1.  Ref: 12887923 Cell, 2003
- Increased kinase activity of p21  [ **PAK3** ]  activated kinase, a specific downstream effector of activated Rac  **Cdc42**  was also observed during the time course of infection.  Ref: 12390349 Cell Microbiol, 2002
- ATX activated  **Cdc42**  and Rac1 and increased complex formation between these small G proteins and p21  [ **PAK3** ]  activated kinase PAK.  Ref: 12482591 FEBS Lett, 2002
- The p21  [ **PAK3** ]  activated kinase PAK homolog, Shk1, is a critical component of a multi functional Ras  **Cdc42**  PAK complex required for viability, polarized growth and cell shape, and sexual differentiation in the fission yeast, Schizosaccharomyces pombe.  Ref: 12764130 J Biol Chem< MedlineTA>J Biol Chem, 2002
